# Lateral position does not cause an interhemicerebral difference of cerebral hemodynamic in healthy adult volunteers

**DOI:** 10.14814/phy2.15685

**Published:** 2023-05-05

**Authors:** Ichiro Kamiya, Chol Kim, Atsuko Kageyama, Atsuhiro Sakamoto

**Affiliations:** ^1^ Department of Anesthesiology Nippon Medical School, Chiba Hokusoh Hospital Chiba Japan; ^2^ Department of Anesthesiology and Pain Medicine Nippon Medical School Tokyo Japan

**Keywords:** cerebral hemispheres, cerebral hemodynamic, lateral position, near‐infrared spectroscopy, regional oxygen saturation

## Abstract

Cerebral perfusion is maintained at a consistent value irrespective of changes in systemic blood pressure or disease‐induced changes in general physical condition. This regulatory mechanism is effective despite postural changes, working even during changes in posture, such as those from sitting to standing or from the head‐down to the head‐up position. However, no study has addressed changes in perfusion separately in the left and right cerebral hemispheres, and there has been no specific investigation of the effect of the lateral decubitus position on perfusion in each hemisphere. Surgery, particularly respiratory surgery, is often performed with the patient in the lateral decubitus position, and since intraoperative anesthesia may also have an effect, it is important to ascertain the effect of the lateral decubitus position on perfusion in the left and right cerebral hemispheres in the absence of anesthesia. The effects of the lateral decubitus position on heart rate, blood pressure, and hemodynamic in the left and right cerebral hemispheres assessed by regional saturation of oxygen measured by near‐infrared spectroscopy were investigated in healthy adult volunteers. Although the lateral decubitus position causes systemic circulatory changes, it may not cause any difference in hemodynamic between the left and right cerebral hemispheres.

## Graphical abstract

The regulatory mechanism of cerebral perfusion is effective despite postural changes, working even during changes in posture. However, there has been no specific investigation of the effect of the lateral decubitus position on hemodynamic in each hemisphere. The effects of the lateral decubitus position on cerebral hemodynamic in the left and right cerebral hemispheres assessed by regional saturation of oxygen measured by near‐infrared spectroscopy were investigated in healthy adult volunteers.

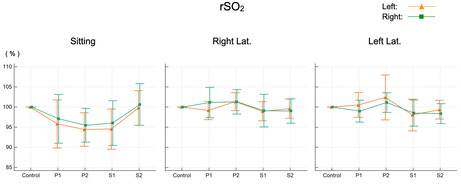

## 1 INTRODUCTION

Cerebral perfusion is maintained at a consistent value irrespective of changes in systemic blood pressure or disease‐induced changes in general physical condition (Armstead, 2016; Claassen et al., 2021). This is also believed to be the case when moving from the supine to the sitting position (Favre et al., 2020; Garrett et al., 2017) when standing up from the sitting position (Favre et al., 2020; Gao et al., 2015; Tachtsidis et al., 2004), and in the head‐down position (Geinas et al., 2012). Changes in systemic circulation are thought to be implicated in this mechanism (Tachtsidis et al., 2004). However, there is no information on the response in terms of changes in cerebral perfusion in the lateral decubitus position. Although cerebral perfusion is known to vary as a result of factors including anesthetics and arterial partial pressure of carbon dioxide (PaCO_2_) (Slupe & Kirsch, 2018; Strebel et al., 1995; Summors et al., 1999), there is no knowledge concerning the intracerebral distribution of perfusion when patients are in the lateral decubitus position, which is used for various procedures, including respiratory surgery.

If the lateral decubitus position does change the distribution of cerebral perfusion, this effect may be greater during anesthesia in this position, and it might have an adverse effect postoperatively on higher brain functions. It is therefore necessary to identify changes in cerebral perfusion as a result of the lateral decubitus position.

In the present study, whether the lateral decubitus position has any effect on cerebral perfusion in the absence of the effect of anesthesia was investigated. To evaluate the cerebral hemodynamic, we measured regional saturation of oxygen (rSO_2_).

## 2 METHODS

Approval for this study (#500) was obtained from the Ethics Committee of Nippon Medical School Chiba Hokusoh Hospital (Inzai, Chiba, Japan) in accordance with the Declaration of Helsinki. Written informed consent was obtained from each participant. To evaluate the effect of posture on cerebral hemodynamic we have observed changes in rSO_2_ at the forehead using the INVOS (Medtronic), which is considered to reflect cerebral hemodynamic. Healthy adult volunteers not younger than 20 years old were enrolled in this study. Candidates who had cerebrovascular diseases, respiratory diseases, and renal diseases were excluded.

Sensing pads for the INVOS (IN Vivo Optical Spectroscopy) 5100C (Medtronic) were placed on both the right and left sides of the forehead to determine rSO_2_. Simultaneously, heart rate (HR) and blood pressure were evaluated. Blood pressure was measured at the right upper arm with an automatic cuff sphygmomanometer through the procedure of this study. When the posture is right decubitus lateral position, right upper arm lies between the body and the bed. In other words, the arm is located under the body. We defined the arm of this position as the dependent arm. The opposite arm is defined as the non‐dependent arm. Thus, the right arm is the dependent arm when the posture is right decubitus lateral position, and it is non‐dependent arm during left decubitus lateral position.

The volunteers were tested in three postures. The postures were changed in the order listed below. Five‐minute breaks between each test position series were given in the supine position at rest.

(1) Sitting position: supine (control)‐sitting position‐supine.

(2) Right lateral position: supine (control)‐right lateral position‐supine.

(3) Left lateral position: supine (control)‐left lateral position‐supine.

At the beginning of each posture series, volunteers were asked to stare at a certain point on the ceiling, remaining at rest for 3 min in the supine position. During the lateral and sitting positions, they were asked to stare at a black cross mark, 10 cm long, on the paper. The data were obtained at the beginning of each posture series after 3 min of rest (control in each figure), immediately after the change to the test position (P1 in each figure), 3 min after the change to the test position (P2 in each figure), immediately after the return to the supine position (S1 in each figure), and 3 min after the return to the supine position (S2 in each figure). The data obtained in the supine position at the beginning of each posture series were used as control values.

Each data value was compared to the control as a ratio, that is, the obtained data value was divided by the control data value, with the control data value regarded as 100. Data are shown as means ± standard deviation. One‐way analysis of variance was used to evaluate the effects of the positions on HR and systolic blood pressure (sBP), and two‐way analysis of variance was used to evaluate the effects of the positions on rSO_2_ at each side of the forehead. Significance was accepted at *p* ≤ 0.05. If significance was observed, Sheffé's post hoc test was used to confirm the significant difference between the control and other measurement points. Statistical analysis was performed using MedCalc (version 20.109; MedCalc Software Ltd., Acacialaan).

## 3 RESULTS

Data were obtained from 20 healthy volunteers (7 males and 13 females). Their mean age was 28.9 ± 8.9 years.

### 3.1 Heart rate

**FIGURE 1:**
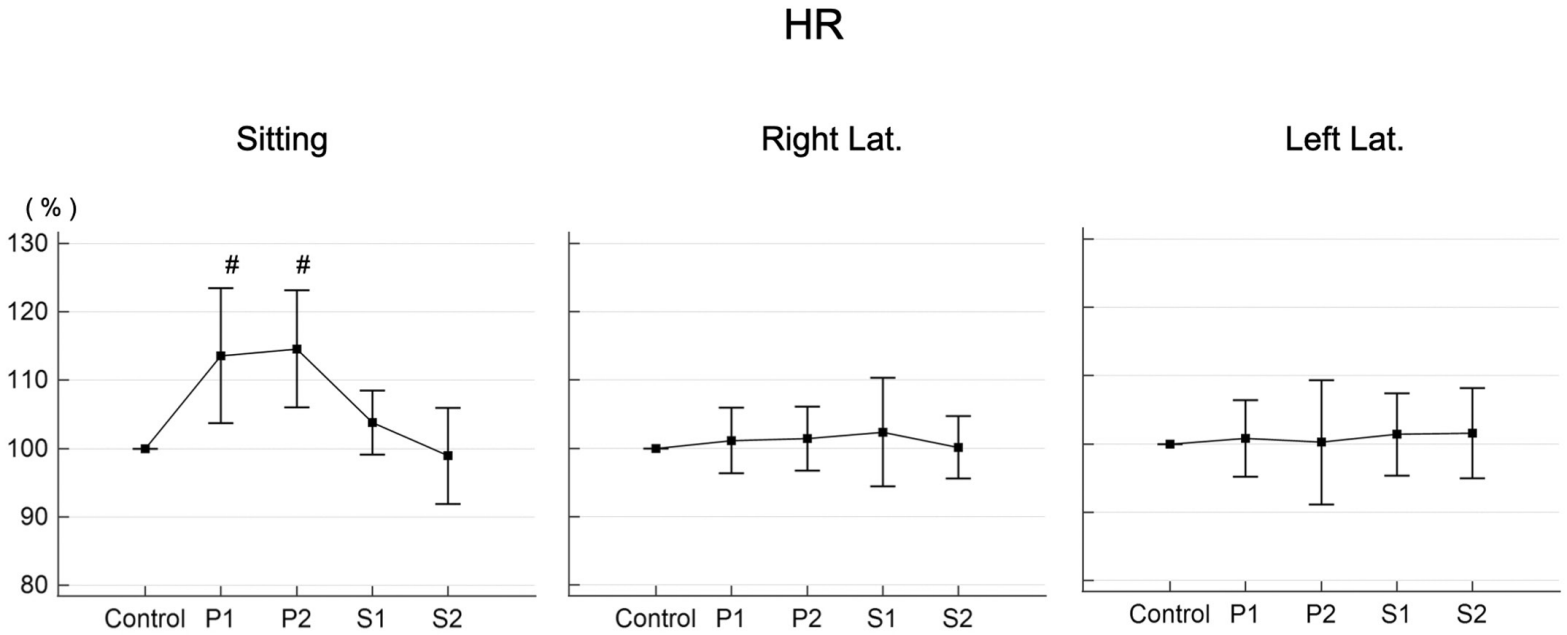
Changes in heart rate (HR). Values at each time point are shown as a percent of control. P1: immediately after changing to the test position. P2: 3 min after changing to the test position. S1: immediately after returning to the supine position. S2: 3 min after returning to the supine position. Right Lat.: right lateral decubitus position. Left Lat.: left lateral decubitus position. #Significant difference compared with the reference value (*p* < 0.05).

The HR increased significantly in the sitting position, but returned to the reference value after 3 min in the supine position (Figure 1, left). There was no significant change in either the right or left lateral decubitus position (Figure 1, middle and right).

### 3.2 Systolic blood pressure

**FIGURE 2:**
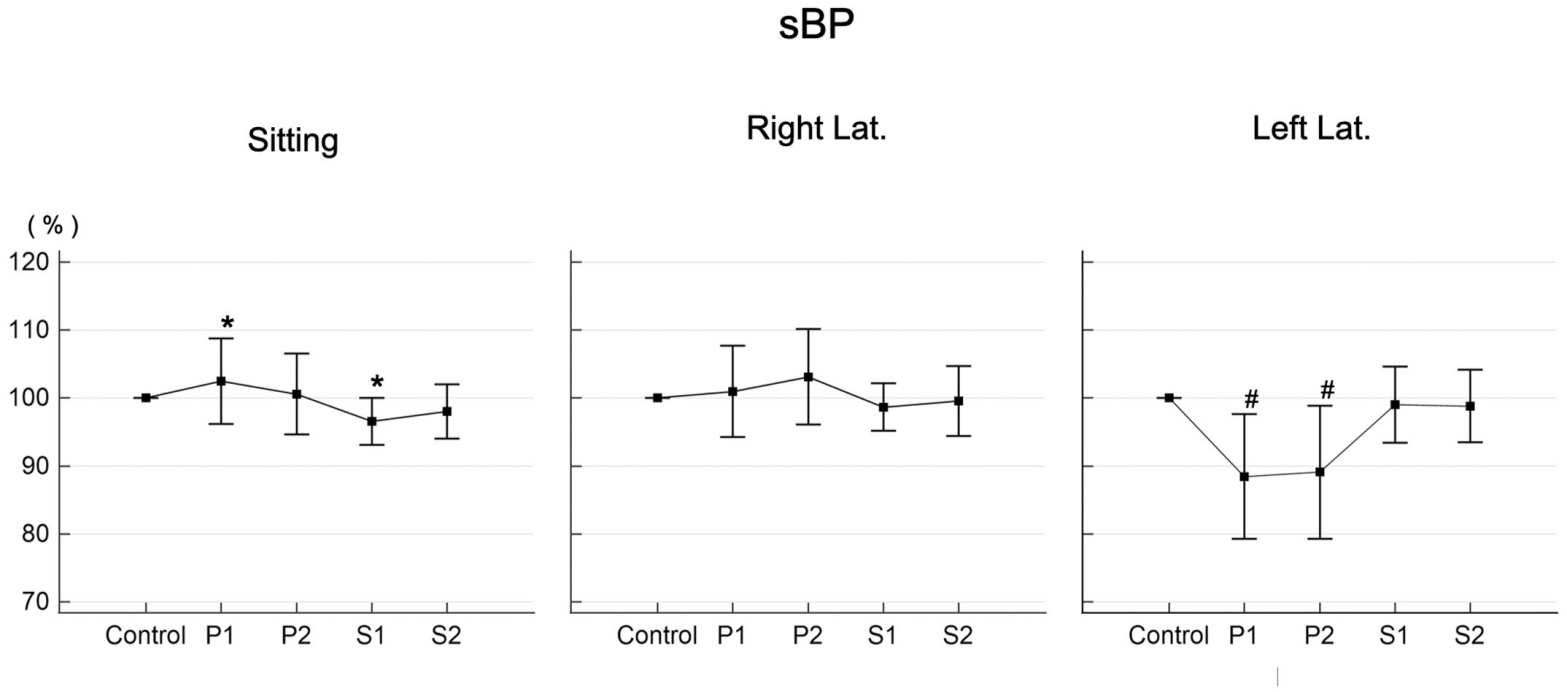
Changes in systolic blood pressure (sBP). Values at each time point are shown as a percent of control. P1: immediately after changing to the test position. P2: 3 min after changing to the test position. S1: immediately after returning to the supine position. S2: 3 min after returning to the supine position. Right Lat.: right lateral decubitus position. Left Lat.: left lateral decubitus position. #Significant difference compared with the reference value (*p* < 0.05). *Significant difference between P1 and S1 (*p* < 0.05).

sBP did not change significantly in the sitting position or the right lateral decubitus position (Figure 2, left and middle). However, it did decrease significantly in the left lateral decubitus position, although it rapidly returned to the reference value in the supine position (Figure 2, right).

### 3.3 rSO_2_

**FIGURE 3:**
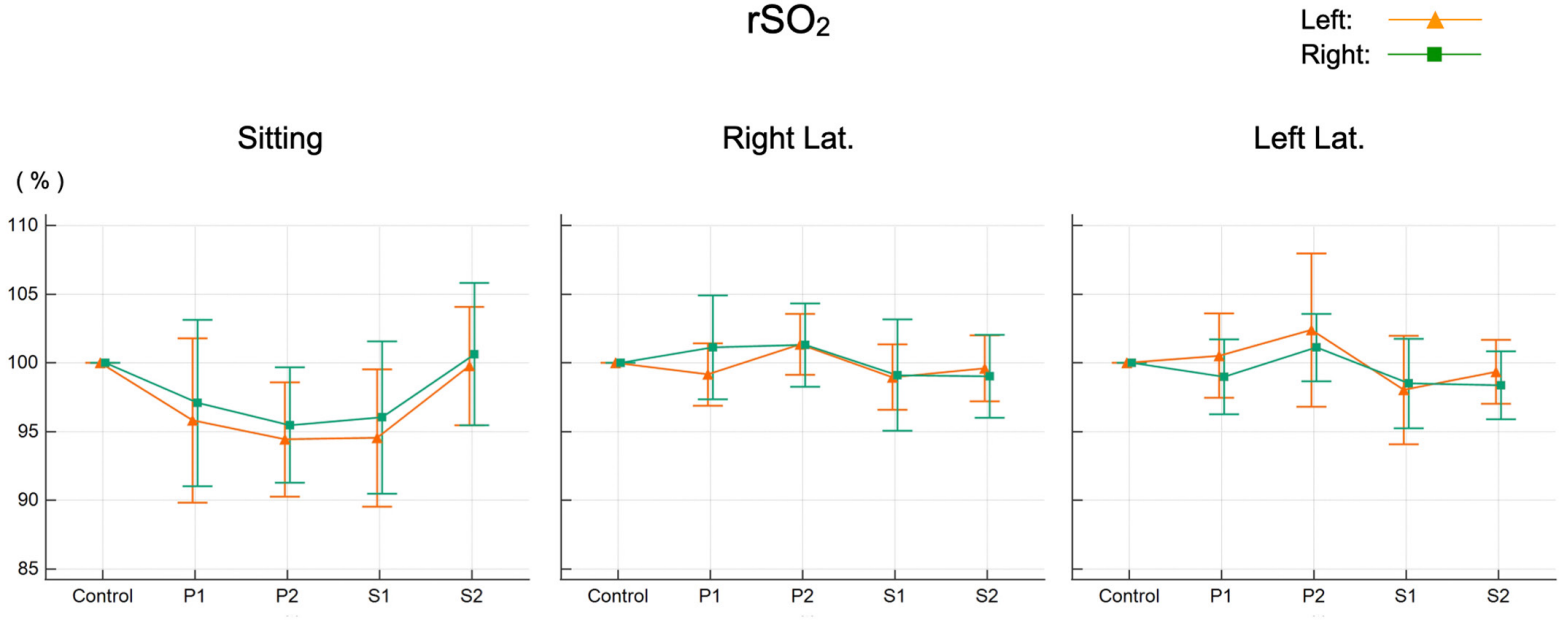
Changes in regional saturation of oxygen (rSO_2_). Values at each time point are shown as a percent of control. P1: immediately after changing to the test position. P2: 3 min after changing to the test position. S1: immediately after returning to the supine position. S2: 3 min after returning to the supine position. Right Lat.: right lateral decubitus position. Left Lat.: left lateral decubitus position.

There was no significant difference in the regional saturation of oxygen between the sitting position and the right and left lateral decubitus positions (Figure 3). In the sitting position, however, it was lower than the reference value on both the left and right sides, and in the right lateral decubitus position, the value on the left tended to be lower (Figure 3, middle), whereas in the left lateral decubitus position, the value on the right tended to be lower (Figure 3, right).

## 4 DISCUSSION

In the present study, the effects of postural changes on cerebral hemodynamic were investigated. In particular, whether the lateral decubitus position causes differences in cerebral hemodynamic between the left and right cerebral hemispheres was examined. The present results suggested that moving from the supine position to the lateral decubitus position does not cause any difference between the left and right sides in cerebral hemodynamic. Although it has been shown that cerebral hemodynamic is maintained during postural changes (Armstead, 2016; Claassen et al., 2021; Favre et al., 2020; Gao et al., 2015; Garrett et al., 2017; Geinas et al., 2012; Tachtsidis et al., 2004), to the best of our knowledge, no previous study has investigated the effect of the lateral decubitus position. It is important to ascertain whether the distribution of cerebral perfusion is normal during operations carried out in the lateral decubitus position, such as respiratory surgery, since measures are required to manage any abnormal distribution.

Although PaCO_2_ was not measured in the present study, the subjects were kept at rest at the time of data acquisition to maintain PaCO_2_ within the range at which it has no effect on cerebral hemodynamic. Following the advice of a psychiatrist, to keep the subjects at rest, they were instructed to stare at a certain point for a fixed amount of time. This was intended to assure the stability of cerebral hemodynamic, including the effect of PaCO_2_.

In the present study, rSO_2_ was selected as a proxy for cerebral hemodynamic measurement, because it is noninvasive and has been shown to reflect cerebral perfusion (Hernandez‐Avila et al., 1995; Kim et al., 2000). It is known that rSO_2_ is the regional oxygen saturation of small vessels (arterioles, venules, and capillary vessels) measured by near‐infrared spectroscopy (NIRS) (Calderon‐Arnulphi et al., 2009). The INVOS 5100C system used in the present study utilizes the spatially resolved spectroscopy technique with two different near‐infrared wavelengths, 730 nm and 810 nm, to calculate the rSO_2_ from the signal data obtained from the deep brain tissue being measured. This has been found to be useful for evaluating the regional status of perfusion and metabolism (Kaminogo et al., 1999; Steiner et al., 2009). During moving the body, such as from sitting to supine position, intracranial blood volume would not be constant, resulting in change of intracranial pressure. This might ruin the reliability of rSO_2_. However, Dummond, JC describes in his review (Drummond, 2019) that there is substantial blood flow reserve that buffers the normal central nervous system against critical blood flow reduction in the face of hypotension, when, probably, mean arterial pressure (MAP) is 40–50 mm Hg at the level of the circle of Willis in a normotensive adult in a vertical posture and 45–55 mm Hg in a supine subject. In our study, all of the subjects are normotensive and did not suffer from critical blood flow reduction nor circulatory blood loss. Thus, it is supposed that the change of intracranial blood volume and pressure causing by the moving of body does not affect the reliability of rSO_2_ in our study.

Cavus et al. (2008) showed experimentally that the time course of treatment for significant decrease in intracranial pressure, mean arterial pressure (MAP), central venous pressure (CVP), and rSO_2_ caused by uncontrolled hemorrhage in swine. In their study, hemorrhage was treated with norepinephrine and small‐volume resuscitation (4 mL/kg). After 5 min of the therapy, rSO_2_ and CVP recovered while MAP did not. This suggests that decrease in intracranial blood volume, caused by losing circulatory blood volume, may impair brain metabolism. Also, Navarro et al. (2012) presented that the decrease in cerebral rSO_2_ is associated with continuous blood loss.

Menke et al. (2004) showed that blood donation of 450 mL in healthy donors caused significant decrease in rSO_2_ and significant increase in cerebral blood volume (CBV). This tapping blood of 450 mL could lead physiological hemodilution. Compensated hemodilution (tapping blood of 300–450 mL, then infusing 1200 mL saline containing 50 g albumin) maintained cerebral metabolic rate of oxygen while cerebral blood flow (CBF) increased (Paulson et al., 1973). However, Ekelund A. et al. showed that hypervolemic hemodilution did not increase CBF (Ekelund et al., 2002). As described the above, hemodilution could influence on not only CBV and CBF, but also on decreasing in viscosity (Paulson et al., 1973) and hematocrit (Bruder et al., 1998; Ekelund et al., 2002) that could have influence on rSO_2_.

The posture change from supine to sitting leads relative decrease in intracranial pressure and increase in CBV theoretically. Also, if hemodilution occurred, brain metabolism could be affected by viscosity and hematocrit. Actually, physiology of brain metabolism is very complicated since there are several factors to be considered. Therefore, further parameters such as viscosity and hematocrit should be measured simultaneously to evaluate the effect of posture change on rSO_2_ precisely.

Park et al. (2009) have shown that rSO_2_ is maintained after the change of posture from supine to Trendelenburg position. In their study, rSO_2_ and mean MAP did not change while CVP increased significantly after the change of position without pneumoperitoneum. After pneumoperitoneum was applied, rSO_2_ and MAP increased as well as CVP. When the posture returned to supine from Trendelenburg position (intracranial blood volume decrease relatively during this procedure, like the posture change to sitting position from supine), CVP decreased in 50% while rSO_2_ remained as increased. This indicates that rSO_2_ could be maintained when CVP decreases associated with the reduction of intracranial blood volume. On the other hand, Tanaka et al. (2019) have shown that Trendelenburg position with pneumoperitoneum did not affect rSO_2_ while CBV increased. When the position returned from Trendelenburg to supine, CBV decreased significantly compared with the control data while rSO_2_ did not changed significantly. Their result suggests that autoregulation of CBF is maintained even if CBV changes during position shifts, indicating that metabolism of brain is preserved, thus rSO_2_ could be a reliable parameter of CBF. There is difference in the effect on rSO_2_ between two studies (Park et al., 2009 and Tanaka et al., 2019). It could be because of difference in pressure level of pneumoperitoneum. Although we cannot confirm this since the data of Park's study is not available, the pressure might have an effect on rSO_2_. This pressure level is important because pneumoperitoneum could change circulatory blood volume, compressing venous vascular bed.

Based on the above, CBF could be kept constant by autoregulation mechanism of CBF even if intracranial blood volume reduced as far as circulatory blood volume is stable. In this condition, we can evaluate cerebral hemodynamic with rSO_2_ as a reliable parameter.

A previous study has shown that rSO_2_ is significantly correlated with jugular venous oxygen saturation and mixed venous oxygen saturation, and its use enables the evaluation of the regional balance between oxygen supply and demand, and perfusion in particular (Kim et al., 2000). Although rSO_2_ cannot provide absolute values for cerebral perfusion, it can be used to evaluate changes in cerebral hemodynamic. In the present study, INVOS sensors were placed on the right and left sides of the forehead. The rSO_2_ values in the left and right cerebral hemispheres could thus be measured independently, enabling changes in the hemodynamic of the frontal lobes of each hemisphere to be evaluated. Theoretically, what was being measured in the present study was the perfusion behind the forehead directly beneath each sensor, which would have been the anterior and middle cerebral arteries. Measuring the rSO_2_ with the INVOS system is thus a valid method of ascertaining whether changing posture from the supine position to the lateral decubitus position gives rise to any left–right differences in changes in cerebral hemodynamic, because it is mainly affected by perfusion in the left and right anterior and middle cerebral arteries.

In the present study, there was an increase in HR in the sitting position (Figure 1, left), but this was a compensatory response by the heart to reduced venous return, which prevented changes in blood pressure (Figure 2, left). This is also thought to help avoid a reduction in cerebral perfusion. Although changes in systemic circulation have been reported to maintain cerebral perfusion during postural changes (Tachtsidis et al., 2004), the change in posture in that study was from sitting to standing. The absence of major changes in the systemic circulation observed in the present study is believed to be because the present protocol focused on changes while lying down. After changing posture to the sitting position, the rSO_2_ tended to decrease in comparison with the reference value, but this change was not significant (Figure 3, left). This suggested that cerebral perfusion is maintained during postural changes, a result consistent with those of previous studies (Favre et al., 2020; Gao et al., 2015; Garrett et al., 2017) that supports the validity of the present study method.

A significant decrease in blood pressure was observed in the left lateral decubitus position (Figure 2, right), but not in the right lateral decubitus position (Figure 2, middle). This was because blood pressure was measured in at the right upper arm through the procedure of this study, and it suggested that left–right differences in systemic circulation may occur in the lateral decubitus position. It is known that there is difference in blood pressures between the right and left arms when they are monitored in the lateral decubitus position (Mostafa et al., 2021; Thomas et al., 2020). The blood pressure of the dependent arm is higher than that of non‐dependent arm by several mm Hg, and non‐invasive blood pressure of the dependent arm correlates well with invasive arterial blood pressure in the lateral decubitus position (Thomas et al., 2020). Thus, it is recommended to measure blood at the dependent arm, not non‐dependent arm in the lateral decubitus position in general. Despite that, we measured blood pressure at the right arm through the procedure of this study to clarify our hypothesis. If rSO_2_ is affected by posture (though actually it is not, shown as in this study), it is expected that difference in rSO_2_ between hemispheres parallels with difference in blood pressures between two arms in the lateral decubitus position. In other words, in lateral decubitus position, if blood pressure of dependent arm is higher than that of non‐dependent arm, it is expected that rSO_2_ of ipsilateral forehead with dependent arm is higher than that of ipsilateral forehead with non‐dependent arm. Or, if rSO_2_ is affected by posture as we assumed, it is expected that the change of rSO_2_ shows different manner from the change of the blood pressure (at the right arm) through this study, since the dependent arm alternates. We supposed that rSO_2_ at the left forehead increases but the blood pressure (at the right arm) decreases in the left decubitus position, while rSO_2_ at right forehead and the blood pressure (at the right arm) increase in the right decubitus position.

In this study, the changes in hemodynamics have been shown to occur at least transiently during postural changes from the supine position to the sitting position and from the supine position to the lateral decubitus position. In particular, differences in blood pressure were shown to occur in the lateral decubitus position, at least between the left and right arms (Figure 2, middle and right). However, there was no significant left–right difference in the measured values of rSO_2_ when changing to the lateral decubitus position (Figure 3). As mentioned above, changes in the systemic circulation are reported to maintain cerebral perfusion during postural changes (Tachtsidis et al., 2004), and in light of the left–right differences in blood pressure in the arms that occur in the lateral decubitus position, the present results suggest the existence of a specific cerebrovascular regulatory mechanism that prevents differences in the distribution of perfusion between the left and right cerebral hemispheres from arising as a result of postural changes.

The present study shows that cerebral hemodynamic is maintained in the lateral decubitus position with no difference between the left and right cerebral hemispheres, since a postural change to the lateral decubitus position does not result in left–right differences in rSO_2_ despite causing left–right differences in systemic circulation.

## 5 CONCLUSIONS

Whether the lateral decubitus position causes differences in cerebral hemodynamic between the left and right cerebral hemispheres was investigated by measuring rSO_2_. Using the study protocol, it was not possible to demonstrate that the lateral decubitus position causes any difference in cerebral hemodynamic between the two cerebral hemispheres. The present study shows that cerebral hemodynamic is maintained during postural changes among the sitting, supine, and lateral decubitus position. Further study is required to evaluate the effect of posture change from supine to lateral decubitus position on rSO_2_ precisely.

## FUNDING INFORMATION

This research did not receive any grants from funding agencies in the public, commercial, or not‐for‐profit sectors.

## CONFLICT OF INTEREST STATEMENT

The authors have no conflict of interest to declare.
